# Anthocyanin-Rich Grape Pomace Extract (*Vitis vinifera* L.) from Wine Industry Affects Mitochondrial Bioenergetics and Glucose Metabolism in Human Hepatocarcinoma HepG2 Cells

**DOI:** 10.3390/molecules23030611

**Published:** 2018-03-08

**Authors:** Nathalia F. F. de Sales, Leandro Silva da Costa, Talita I. A. Carneiro, Daniela A. Minuzzo, Felipe L. Oliveira, Lourdes M. C. Cabral, Alexandre G. Torres, Tatiana El-Bacha

**Affiliations:** 1 Instituto de Química, Laboratório de Bioquímica Nutricional e de Alimentos, Universidade Federal do Rio de Janeiro, Rio de Janeiro, RJ 21941902, Brazil; torres@iq.ufrj.br; 2 Instituto de Bioquímica Médica, Universidade Federal do Rio de Janeiro, Rio de Janeiro, RJ 21941902, Brazil; silvadacostta@gmail.com; 3 Instituto de Nutrição Josué de Castro, Laboratório de Estudos com Bioativos de Alimentos e Metabolismo Energético, Universidade Federal do Rio de Janeiro, Rio de Janeiro, RJ 21941902, Brazil; talita.tiac@gmail.com (T.I.A.C.); daniela.minuzzo@gmail.com (D.A.M.); 4 Instituto de Ciências Biomédicas, Universidade Federal do Rio de Janeiro, Rio de Janeiro, RJ 21941902, Brazil; felipe@histo.ufrj.br; 5 EMBRAPA Agroindústria de Alimentos, Guaratiba, Rio de Janeiro, RJ 21941902, Brazil; lourdes.cabral@embrapa.br

**Keywords:** grape pomace, anthocyanins, polyphenols, bioactivity, cancer, mitochondrial bioenergetics, glucose metabolism, in vitro, HepG2 cells

## Abstract

Cancer cells demand high ATP provisions to support proliferation, and targeting of energy metabolism is a good strategy to increase their sensitivity to treatments. In Brazil, wine manufacture is expanding, increasing the amount of pomace that is produced. We determined the phenolic composition and antioxidant properties of a dark skin Grape Pomace Extract and its effects on metabolism and redox state in human hepatocarcinoma HepG2 cells. The material and the methods used represented the industrial process since pomace derived from white wine production and the extract concentrated by pilot plant scale reverse osmosis. Grape pomace extract was rich in polyphenols, mainly anthocyanins, and presented high antioxidant capacity. Short-term metabolic effects, irrespective of any cytotoxicity, involved increased mitochondrial respiration and antioxidant capacity and decreased glycolytic metabolism. Long-term incubation was cytotoxic and cells died by necrosis and GPE was not toxic to non-cancer human fibroblasts. To the best of our knowledge, this is the first report to characterize pomace extract from white wine production from Brazilian winemaking regarding its effects on energy metabolism, suggesting its potential use for pharmaceutical and nutraceutical purposes.

## 1. Introduction

Cancer remains a leading cause of death worldwide despite considerable advances in basic research and clinical trials. Additionally, the side effects of conventional therapies contribute to decrease the patients’ quality of life and the need for the development of safe and effective alternative therapies is high. Cancer cells demand higher ATP provisions to support accelerated proliferation and associated processes when compared to non-cancer cells and targeting energy metabolism appears to be a good strategy to increase their sensitivity to treatments [1].

An ever-increasing amount of data shows that polyphenols present anti-cancer effects due to their anti-inflammatory, anti–angiogenic and redox properties [2]. The mechanisms by which polyphenols induce cancer cells death involve, directly and indirectly, the regulation of mitochondrial function and energy metabolism [3,4]. Resveratrol, quercetin, catechin, curcumin, oroxylin are some polyphenols that were shown to affect mitochondrial function by regulating the activity of apoptotic and electron transport chain proteins and mitochondrial membrane potential [4,5]. These findings clearly highlight the potential use of polyphenols as chemotherapeutic agents targeting energy metabolism. Unfortunately, most of the biological activities of polyphenols reported to date are mainly theoretical because direct evidence of these mechanisms occurring in vivo has not been shown. One explanation for that is the synergistic or additive effects of polyphenols when they are present in whole extracts from food or food residues, as opposed to the effects of purified compounds [6,7].

In Brazil, wine manufacture is expanding and in 2015 wine production was approximately 250 million L [8]. Grape pomace is the major wine industry residue and it raises environmental issues concerning proper destination, especially because of the high volume of waste produced (20 kg pomace/100 L of white wine) [9]. Unfortunately, the use of grape pomace for agriculture purposes is limited due to its high content of polyphenols including flavonoids, phenolic acids, stilbenes and tannins that are potentially toxic to the soil microbiota [9,10,11]. The development of bioactive products for food and pharmaceutical industries might add value and increase industrial destination of grape pomace. This potential industrial application might have beneficial effects to human health while contributing to the proper destination of grape pomace and to decrease its environmental impact.

Bioactivity, metabolism and antioxidant properties of grape pomaces vary as a function of the raw material, particularly grape cultivar and vintage and the winemaking method [10]. Therefore, studying grape pomace bioactivity in human cells, and how it relates to its antioxidant potential and its chemical composition are necessary to assess eligibility for future product development. In this study, we determined the phenolic composition and antioxidant properties of a dark skin grape (Pinot noir) pomace extract, concentrated by membrane technology, as well as its effects on mitochondrial function, glucose metabolism and redox state in human hepatocarcinoma HepG2 cells. Importantly, the material and the methods applied in this study are representative of the industrial process since the red grape pomace used was derived from industrial white wine production and the extraction and concentration processes were carried out in a pilot plant scale.

## 2. Results

### 2.1. Pinot Noir Grape Pomace Extract (GPE) Is Highly Concentrated in Phenolic Compounds and Anthocyanins

**Figure 1:**
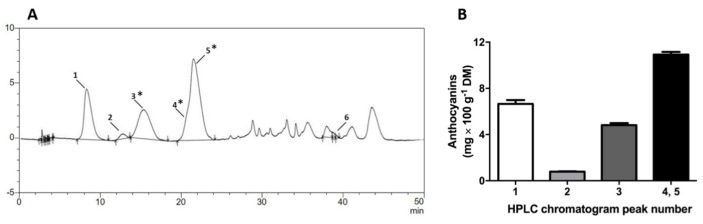
Grape (Pinot noir) pomace extract (GPE) anthocyanins analysis by HPLC-PDA. (**A**) Representative HPLC chromatogram of GPE, showing the major anthocyanins that were identified, indicated by peak numbers and; (**B**) contents of anthocyanins in GPE (chromatographic peaks 1–5); Peak identities: 1, Delphinidin-3-glucoside; 2, Cyanindin-3-glucoside; 3 *, Petunidin-3-glucoside; 4 *, Peonidin-3-glucoside; 5 *: Malvidin-3-glucoside; 6: Malvidin. * Tentatively identified as anthocyanin, based on peak spectra in PDA detector.

GPE was highly concentrated in phenolic compounds (Figure 1 and Table 1), taken from the contents of total phenolic, total flavonoids, total and monomeric anthocyanins, and individual anthocyanins identified by HPLC-PDA. The high phenolic content of GPE was reflected by its high antioxidant capacity determined by ABTS, ORAC and FRAP assays (Table 1). The major phenolic compounds in the GPE were anthocyanins. The following six anthocyanins were identified: delphinidin-3-*O*-glucoside, cyanidin-3-*O*-glucoside, petunidin-3-*O*-glucoside, peonidin-3-*O*-glucoside, malvidin-3-*O*-glucoside and malvidin. Malvidin-3-*O*-glucoside was the major anthocyanin identified in GPE. Chromatographic peaks numbered 3, 4 and 5 (Figure 1A) were tentatively identified, based on reversed-phase chromatographic behavior of anthocyanins and PDA-spectra (Supplementary Materials Figures S1–S4) [11]. The Photodiode Array (PDA)-Vis-spectra of the unresolved peaks 4 and 5 were obtained respectively from the beginning (peak 4) and end (peak 5) within the time interval corresponding to these peaks, and had different λmax (519 and 527, respectively), which were compatible with peonidin-3-glucoside and malvidin-3-glucoside (Supplementary Materials Figures S1–S4).

### 2.2. GPE Induced Alterations in HepG2 Morphology

**Figure 2:**
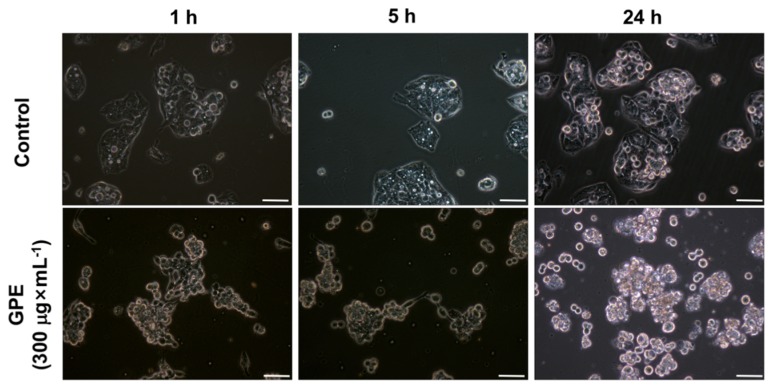
Grape pomace extract (GPE) induced morphological changes in HepG2 cells. The cells were incubated with ethanol (control) or 300 μg × mL^−1^ GPE for the indicated times and photographed under bright field using the documentation system of inverted microscope Nikon TS100. Bar = 20 μm.

Incubation of HepG2 cells with GPE resulted in several morphological changes, particularly when incubated with 300 μg × mL^−1^ (Figure 2). HepG2 grows in clusters and do not form a monolayer (Figure 2, control), which is typical of a proliferative cell line that does not suffer contact inhibition. Following 1 h incubation with GPE, we observed that HepG2 cells started to present a more rounded shape when compared to control cells incubated with ethanol (same concentration as in the GPE), particularly those that grew on the top of the layers. This possibly indicated a decreased cellular adherence. After 5 h of incubation with GPE, a significant proportion of the cells rounded up and at 24 h incubation, most HepG2 cells were detached from the flasks. Additionally, at 24 h, detached HepG2 cells were smaller than adherent cells, indicating significant cell death. The early (1 h) morphological alterations induced by GPE might be related with decreased HepG2 cell viability after medium- and long-term GPE treatment.

### 2.3. Pinot Noir Grape Pomace Extract (GPE) Was Cytotoxic to HepG2 Cells

**Figure 3:**
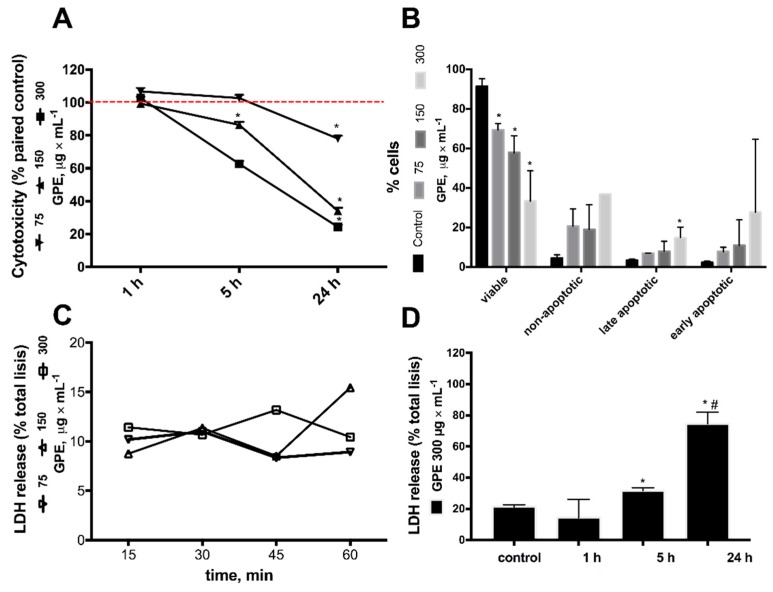
Cytotoxicity of grape pomace extract (GPE) on human hepatocarcinoma cell line HepG2. GPE induced a time and concentration dependent cytotoxic effect on HepG2 cells. (**A**) Cytotoxicity was evaluated by the MTT assay after 1, 5 and 24 h incubation with 75 (▼); 150 (▲); 300 (■) μg × mL^−1^ GPE. Results are expressed relative to their respective paired control (ethanol as vehicle). Red line indicates viability of control cells (100 %); (**B**) quantitation of viable, non-apoptotic (Annexin^−^/PI^+^), late apoptotic (Annexin^+^/PI^+^) and early apoptotic (Annexin^+^/PI^−^) HepG2 cells evaluated by flow cytometry after 24 h incubation with GPE; control (black bars); medium gray, dark gray and light gray represents 75, 150 and 300 μg × mL^−1^ GPE, respectively; (**C**) LDH release was evaluated up to 1 h incubation with 75 (∇); 150 (Δ); 300 (☐) μg × mL^−1^ GPE; (**D**) Cytotoxicity was evaluated by LDH release to the culture medium, after incubation with 300 μg × mL^−1^ GPE for the indicated times. Results are expressed relative to maximal cell lysis after detergent addition. (**A**) * Significantly different from their respective control (ethanol; *p* < 0.05); (**B**) * Significantly different from control (ethanol; *p* < 0.05) cells within the respective group; (**D**) * Significantly different from control (ethanol; *p* < 0.001) and 1 h incubation with GPE; # Significantly different from 5 h incubation with GPE (*p* < 0.001).

To investigate further the morphological alterations in HepG2 cells promoted by GPE, different assays were used to access cytotoxicity (Figure 3 and Supplementary Materials Figure S1). IC_50_ for GPE was in the range of 200 μg × mL^−1^ (Supplementary Materials Figure S5) and GPE promoted a concentration and time dependent decrease in metabolic activity of HepG2 cells (Figure 3A). It can be seen that 1 h incubation with GPE did not change the metabolic activity when compared to control cells, whereas at 24 h, metabolic activity was reduced by 80% with 150 and 300 μg × mL^−1^ GPE. The effects of GPE on BEAS human fibroblast cell line metabolic activity was also evaluated for comparison and, in contrast to HepG2 cells, a feeble 10% decrease was detected after 24 h incubation with 300 μg × mL^−1^ (Supplementary Materials Figure S6). Flow cytometry was used to assess the quantity of dead cells after 24 h incubation with GPE (Figure 3B). The percentage of viable cells (Annexin V^−^/PI^−^) decreased as a function of GPE concentration, where a 30%, 40% and 70% decrease was observed after incubation with 75, 150 and 300 μg × mL^−1^ GPE. These results were in agreement with the IC_50_ calculated from the MTT reduction assay (Supplementary Materials Figure S5). Inversely, the percentage of non-apoptotic (Annexin V^-^/PI^+^), late apoptotic (Annexin V^+^/PI^+^) and early apoptotic (Annexin V^+^/PI^−^) cells showed a clear tendency to increase as GPE concentration increased. Although most cells were Annexin V^−^/PI^+^, statistical significance was reached only in the late apoptotic (PI^+^/Annexin V^+^) by 300 μg × mL^−1^ GPE. Cytotoxicity of GPE was also accessed by the degree of LDH release to the culture media (Figure 3C,D). As observed in the MTT assay, LDH release remained as low as 15% after 1 h incubation with all concentrations of GPE, as observed in HepG2 cells incubated with ethanol. However, after 24 h incubation, cell lysis was approximately 80% (Figure 3C,D). These cytotoxic effects agree with the morphological alterations induced by GPE in HepG2 cells at 24 h incubation (Figure 2).

### 2.4. GPE Increased Mitochondrial Respiration and Antioxidant Capacity in HepG2 Cells

**Figure 4:**
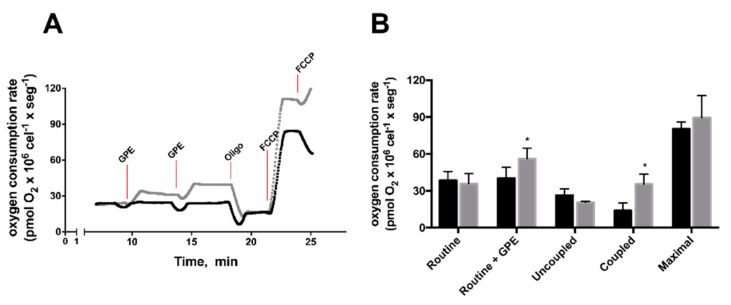

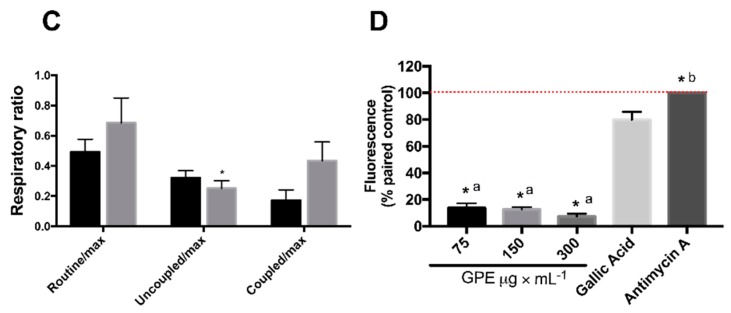
Grape pomace extract (GPE) increased mitochondrial respiration and antioxidant capacity in HepG2 cells: (**A**) Representative traces of oxygen consumption in control (black trace) and GPE-treated (gray trace) cells. Routine respiration was evaluated after 2 consecutive additions of GPE (300 μg × mL^−1^, each), following the addition of olygomycin (1 μg × mL^−1^), to measure oxygen consumption uncoupled to ATP synthesis and the ionophore FCCP (250 nmol × L^−1^), to induce maximal respiration; (**B**) Control and GPE incubated HepG2 average routine respiration, uncoupled and coupled respiration to ATP synthesis (measured after oligomycin addition) and FCCP-induced maximal respiration, calculated from the traces shown in A; (**C**) Respiratory ratios, calculated from data presented in B; (**D**) Antioxidant capacity of HepG2 cells incubated with GPE: Cells were incubated in the absence or presence of GPE at the indicated concentrations for 1 h. Fluorescence was expressed relative to control, normalized by maximum fluorescence, in the presence of Antimycin A (2 μg × mL^−1^). Gallic Acid was used as a standard antioxidant molecule. * Oxygen consumption rates and respiratory ratio different from control HepG2 (*p* < 0.05); * Fluorescence different from Antimycin A (*p* < 0.0001); Distinct letters: Fluorescence different from Gallic Acid (*p* < 0.01). Red line indicates maximum fluorescence (100%) after Antimycin A addition.

Since GPE cytotoxicity was observed from 1 h onwards, the next set of experiments was designed to address whether alterations in HepG2 energy metabolism preceded cell death. Therefore, the effects of short-term incubation of GPE (up to 1 h, 300 μg × mL^−1^) were evaluated on mitochondrial bioenergetics, antioxidant capacity, glycolytic flux and metabolites contents. GPE increased routine respiration by 30% and coupled respiration by 50% (Figure 4A,B). Routine flux control ratio (Routine/max), which expresses how close routine respiration works from maximal respiratory capacity, and the fraction of oxygen consumption rate utilized for ATP synthesis (Coupled/max) slightly increased in GPE treated cells, although not statistically significant (Figure 4C). On the other hand, the uncoupled flux control ratio (Uncoupled/max), which reflects the extent of intrinsic uncoupling, was decreased by 30% in GPE incubated cells (Figure 4C). Because the increase in mitochondrial function observed in GPE-treated HepG2 cells could alter cellular redox state, we next sought to evaluate the effects of GPE on reactive oxygen species content on HepG2 cells (Figure 4D). In this experiment, cells were challenged with antimycin A, which is an inhibitor of respiratory chain complex III, known to increase intracellular ROS production. Therefore, the resulting effects would express the regulation of the antioxidant capacity by GPE in HepG2 cells. Maximal oxidation of H_2_DCFDA measured in the presence of antimycin A (and in the absence of GPE) was the reference fluorescence value. One-hour incubation with all GPE concentrations promoted a significant 85–90% decrease in H_2_DCFDA oxidation. This decreased H_2_DCFDA fluorescence reflects reduced ROS accumulation in HepG2 cells incubated with GPE, which might be associated with an increase in mitochondrial respiration.

### 2.5. Grape Pomace Extract Decreased Glycolytic Flux and Affected Intracellular Metabolites in HepG2 Cells

**Figure 5:**
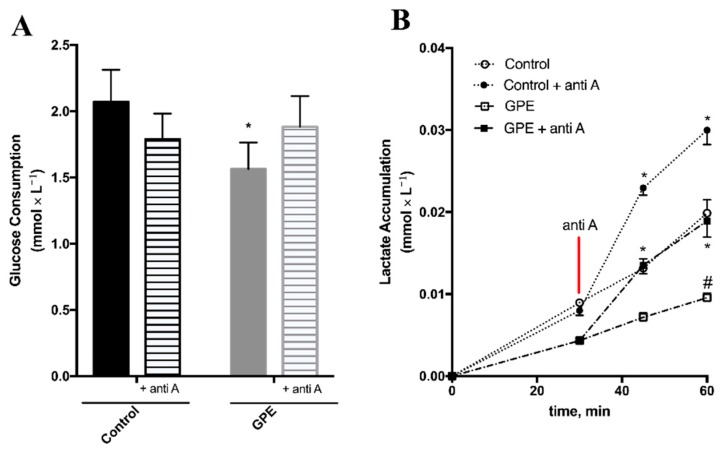
GPE decreased glucose uptake and glycolytic flux in HepG2 cells. HepG2 cells were treated for 1 h with 300 × mL^−1^ GPE after which culture medium was replaced by 5 mM ^13^C-U-glucose DMEM to measure (**A**) glucose uptake and (**B**) lactate accumulation in the culture medium. Aliquots of the supernatant were collected every 15 min, up to 60 min. Antimycin A (Anti A), an inhibitor of mitochondrial complex III, was added after 30 min, to evaluate maximum lactate efflux and glycolytic capacity. ^13^C glucose disappearance from and ^13^C lactate efflux to the culture media were measured by one dimension ^13^C-NMR spectroscopy. *n* = 4 independent experiments, * *p* < 0.05, compared to HepG2 cells without antimycin A; # *p* < 0.05 compared to control HepG2 cells, without GPE.

The effects of GPE on mitochondrial respiration suggested that energy metabolism is an early target of GPE polyphenols. These metabolic effects of GPE on HepG2 were further investigated by measuring glucose consumption and lactate accumulation in the culture medium, as proxies of the glycolytic flux, and the results confirmed that GPE affected oxidative pathways in HepG2 cells. In these experiments antimycin A was used to force cells to rely solely on glycolysis and to evaluate maximal glycolytic capacity. GPE incubation decreased glucose consumption by 25% in HepG2 cells. In control cells, which are highly glycolytic, antimycin A incubation did not change glucose consumption, but in the more oxidative GPE-treated HepG2 cells, inhibition of respiration by antimycin A increased glucose consumption by 20% (Figure 5A).

Lactate accumulation increased as a function of time in both control and GPE-treated cells (Figure 5B; open symbols). However, in GPE-treated cells, lactate accumulation was 50% lower than that of control cells at 60 min. The addition of antimycin A significantly increased the amount of lactate accumulated in the culture media of control and GPE-treated cells (Figure 5B; closed symbols), but this increase was 37% lower in GPE-treated cells when compared to controls. These results show that, even though inhibition of respiration by antimycin A increased glucose consumption and glycolysis is functional in HepG2 cells incubated with GPE, total glycolytic capacity was decreased after GPE incubation.

**Figure 6:**
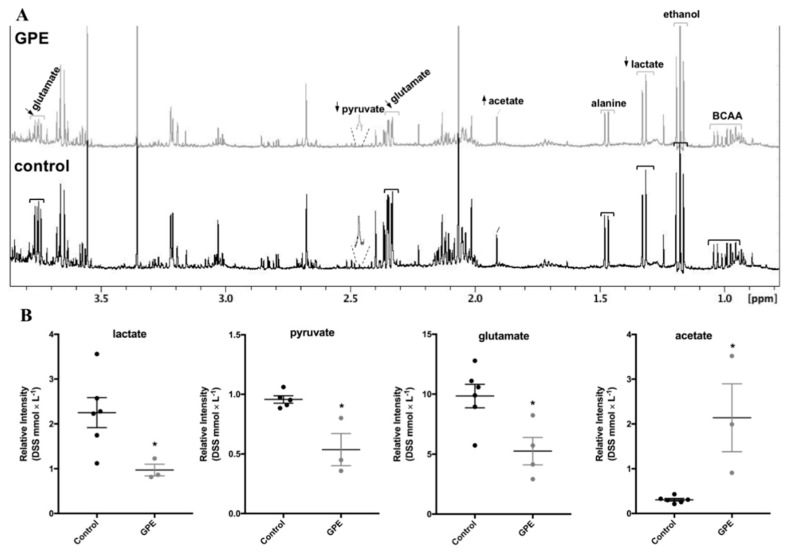
GPE affected intracellular metabolites in HepG2 cells. HepG2 cells were treated for 1 h with 300 × mL^−1^ GPE after which intracellular metabolites were evaluated in a protein-free cellular extracts by one dimension ^1^H-NMR spectroscopy. (**A**) Representative ^1^H-NMR spectrum, showing the decrease μ(↓) or increase (↑) in metabolites content in GPE compared to control HepG2 cells; (**B**) Relative intensity of lactate, pyruvate, glutamate and acetate peaks in HepG2 cells. *n* = 4 independent experiments, * *p* < 0.05, compared to control cells. BCAA: branched-chain amino acids; ethanol used as vehicle.

The increase in mitochondrial respiration and the decrease in glycolytic capacity promoted by GPE affected intracellular metabolites in HepG2 cells (Figure 6). Lactate and pyruvate contents were decreased in HepG2 cells incubated with GPE, in agreement with the decrease in lactate accumulation in the culture media (Figure 5B). Additionally, glutamate content was decreased in GPE-treated cells, which might indicate an increased use of this amino acid as a respiratory substrate, because cellular respiration, particularly coupled respiration, increased after addition of GPE (Figure 4B). On the other hand, GPE induced a significant increase in acetate content, which might be related to a decrease use in this compound.

## 3. Discussion

In the present work, we showed that GPE from wine industry presented high contents of polyphenols, the majority of which being anthocyanins, and high antioxidant capacity. The short-term metabolic effects of GPE on HepG2 cells were irrespective of any cytotoxicity and comprised an increase in mitochondrial respiration and antioxidant capacity and a decrease in glycolytic capacity. Long-term incubation of HepG2 cells with GPE induced significant cytotoxic effects and cell death mainly by necrosis and GPE was not toxic to non-cancer human fibroblasts cells, which highlights its potential use for pharmaceutical and nutraceutical purposes. To the best of our knowledge, this is the first report of pomace extract from industrial white winemaking of red grapes, being characterized regarding its effects on energy metabolism.

Total content and profile of phenolic compounds of grape pomace are highly variable and depend on cultivar and vintage, and on the practices adopted in winemaking [12]. The chemical composition of the GPE used in the present study agrees with previous works, where anthocyanins were the major polyphenols in grape pomace [12,13,14,15,16]. Additionally, the same anthocyanins we identified were previously reported in grape pomace, despite natural variations in their content [10,17]. Total phenolic and anthocyanins contents in red wine grape pomace from Brazilian winemaking industry were previously shown to vary depending on grape cultivar, and Cabernet Sauvignon was the variety with the richest pomace extract [15]. The values we found for total phenolic compounds of Pinot Noir pomace extract in the present work were 35% lower and total anthocyanins content was very similar to those found for Cabernet Sauvignon pomace [14,15]. However, the process of making white wine extracts low amounts of polyphenols from the grape skin into the wine, and therefore tend to result in a richer pomace. Consistently, we found that contents of total phenolics were 10-fold higher and that of anthocyanins were 4-fold higher in the present study compared to Pinot Noir residue from red winemaking in Brazil [14].

In agreement with its high polyphenols content, GPE presented high antioxidant capacity. The application of reverse osmosis and the fact that the extract was derived from pomace of white winemaking process, made it possible to produce a GPE with much higher antioxidant capacity potential when comparing to Pinot Noir skin residues and Cabernet Sauvignon pomace extracts from Brazilian winemaking [14,15]. The membrane technology used in this study concentrated anthocyanins and total antioxidant capacity of GPE by 9-fold [18]. Since the solvent and the extraction conditions were done to maximize the extraction of polyphenols in general, particularly anthocyanins, from grape pomace, we reckon that these compounds accounted for most of the bioactivity of GPE described in the present work.

In the present study, the human hepatocarcinoma cell line HepG2 was used to evaluate the bioactivity of grape pomace. Other authors have shown that HepG2 cells viability was affected by grape skin but not grape seed extracts, indicating that bioactivity in grape pomace extract would come from compounds in the skin [17]. Additionally, the effects of isolated polyphenol compounds were also investigated in HepG2 cells. Quercetin and the flavone hispidulin were found to decrease HepG2 viability in much higher and longer incubation periods [19,20,21]. The fact that we found much more pronounced cytotoxic effects of GPE in HepG2 cells compared to purified compounds indicates that GPE compounds might present synergistic or additive effects, as previously suggested [7]. Indeed, it was shown recently that white grape pomace extract, rich in flavonoids and phenolic acids, also presented cytotoxic and anti-proliferative effects on Caco-2 cells [22].

The most important set of results we presented in this study was the short-term effects of GPE on mitochondrial function, irrespective of the effects on HepG2 cells viability. GPE increased mitochondrial respiration, particularly the fraction of oxygen consumption used to drive ATP synthesis and these effects were accompanied by an increase in cellular anti-oxidative capacity.

This anti-oxidative effect of GPE was significant even when compared to the effects of gallic acid, a reference anti-oxidative molecule (Figure 4D). We reckon the modulation of mitochondrial bioenergetics and the anti-oxidative effects triggered the cytotoxic effects observed after 24 h incubation with GPE. One possible explanation for that is the signaling role played by reactive species in cancer cells, which might seem important for these cells to proliferate [23] and it appears that the decrease in the reactive species, due to GPE increase in mitochondrial respiration, paved the way for the cytotoxic effects observed at longer incubation periods with the extract.

The study on the effects of flavonoids on mitochondrial function is now growing [4]. Anthocyanins were the major flavonoids found in GPE produced in the present study (Figure 1) and possibly the biological actions of anthocyanins were the major determinants of the metabolic effects described in the present work. The effects of anthocynin-rich extracts (*Vaccinium myrtillus* L.) on mitochondrial bioenergetics have been evaluated in isolated heart mitochondria where an increase in uncoupled respiration followed by a decrease in H_2_O_2_ production were observed. Isolated malvidin-3-*O*-glucoside, malvidin-3-*O*-galactoside and cyanidin-3-*O*-galactoside, major anthocyanins in the GPE produced in our study, on the other hand, did not present any effects [24]. In contrast, in a model of ischemia-reperfusion stress, it was shown that isolated delphinidin-3-*O*-glucoside and cyanidin-3-*O*-glucoside protected isolated heart mitochondria from oxidative damaged by preventing the oxidation of complex I, by increasing mitochondrial respiration coupled to ATP synthesis and by decreasing cytochrome *c* oxidation, blocking the apoptotic cascade [25]. According to our results, GPE increased mitochondrial coupled respiration and decreased ROS production. Therefore, due to chemical structural differences and whether anthocyanins are isolated or presented in a complex mixture, they seem to present different cellular bioactivity and possibly synergistic or additive effects. Additionally, it might be speculated that different signaling pathways might be activated, depending on the metabolic activity of the cells exposed to the anthocyanins.

The fact that GPE decreased glucose consumption and lactate efflux indicates that this extract promoted a less glycolytic phenotype, which was associated with a decrease in cell proliferation. These effects were previously associated with features of differentiation in cancer cells, as shown by our research group [26,27]. The GPE-induced decrease in anaerobic glycolysis was followed by an increase in oxidative pathways, as indicated by increased oxygen consumption. Indeed, decreased intracellular glutamate in GPE-treated HepG2 cells (Figure 6B) might have reflected an increase in mitochondrial glutamine oxidation. Published scientific data are still scarce regarding the overall effects of polyphenols on glucose metabolism, and it was shown recently that an anthocyanin rich strawberry extract decreased glycolysis and induced apoptosis in human uterine leiomyoma cells, a model of benign uterine tumors [28]. Interestingly, we also observed a decrease in intracellular pyruvate and lactate after GPE incubation (Figure 6B), adding to the metabolic effects of GPE polyphenols on decreasing glucose uptake and glycolytic flux of HepG2 cells. Additionally, the increase in acetate content (Figure 6B) might also be related to the overall decreased utilization of carbons induced by GPE polyphenols. Possibly, in GPE-treated cells, less acetate is being used in acetylation reactions involved in chromatin remodeling and regulation of gene transcription. Although this hypothesis needs to be addressed, it would be one possible mechanism by which polyphenols induced HepG2 cells death after long incubation period.

## 4. Materials and Methods

### 4.1. Chemicals and Reagents

T2,2′-azinobis-(3-ethylbenzothiazoline-6-sulfonic acid (ABTS), 6-hydroxy-2,5,7,8-tetramethylchroman-2-carboxylic acid (Trolox), 2,2′-azobis (2-amidinopropane) dihydrochloride (AAPH), fluorescein disodium salt, Folin-Ciocalteu reagent, Gallic acid, catechin, 5-(and-6)-chloromethyl-2′,7′-dichlorodihydrofluorescein diacetate, acetyl ester (CM-H2DCFDA), ethanol, acetone, isopropanol, hydrochloric acid (HCl) were purchase from Sigma-Aldrich. 3-(4,5-dimethylthiazol-2-yl)-2,5-diphenyltetrazolium bromide (MTT), antimycin A, Dulbecco Modified Eagle Medium (DMEM) and Phosphate buffer saline (PBS) were purchase from Invitrogen (Waltham, MA, USA).

### 4.2. Grape Pomace

Grape pomace from Pinot Noir was derived from white wine production and obtained from a large-scale winemaking industry in Bento Gonçalves, Rio Grande do Sul, Brazil, through a cooperation with the Embrapa Grape and Wine unit. Grape pomace was extracted and concentrated in a reverse osmosis plant at Embrapa as described previously [29]. Briefly, before extraction, grape pomace was re-hydrated for 1 h, in a 2:1 (*w*/*v*) pomace:water ratio, at 30 °C. Raw extract was produced by mixing hydrated pomace with 30% acidified ethanol (pH 4.0), in a 1:9 (*w*/*v*) pomace: solvent ratio, at 50 °C for 2 h, with mechanical mixing followed by sieving through a 150 μm-pore nylon membrane by centrifugation (38× *g*), in order to separate the liquid extract. The raw extract was then concentrated 8-fold by reverse osmosis, in a pilot-plant, by applying 60.0 Bar to the membrane, with 30% recirculation of the concentrated affluent at 700 L × h^−1^. Ethanol final concentration was kept at 30% in the concentrated extract. The concentrated grape pomace extract was filtered in a 0.45 µm membrane, and stored at −20 °C until analysis and use in cell culture experiments.

### 4.3. Chemical Assays

#### 4.3.1. ABTS Radical TEAC Assay

Trolox equivalent antioxidant capacity (TEAC) assay is based on the capacity of a sample to reduce the ABTS radical (ABTS^•+^) compared with a reference antioxidant standard (Trolox^®^). Calibration curve was in the range 100 to 800 µmol × L^−1^ Trolox^®^. Absorbance was monitored at 734 nm in a microplate reader (Victor 3; Perkin Elmer, (Waltham, MA, USA), and the values were expressed as µmol of Trolox^®^ antioxidant equivalent per g of sample.

#### 4.3.2. ORAC Assay

Oxygen radical antioxidant capacity (ORAC) assay was adapted from [30]. ORAC assay kinetically measures peroxyl radical scavenging activity of the sample using Trolox^®^ as the antioxidant standard. Fluorescein was used as the fluorescent probe and the peroxyl radicals were generated from AAPH in phosphate buffer, pH 7.4. Calibration curve was in the range 20 to 120 µmol × L^−1^ Trolox^®^. In a 96-well plate, sample or standard plus fluorescein were added, in triplicate. The plate was placed in a microplate reader (Victor 3; Perkin Elmer, (Waltham, MA, USA) and the reaction was started by addition AAPH to the wells with an automatic sampler. Fluorescence was recorded every 5 min for 1 h (λ_ex_ = 485 nm and λ_em_ = 535 nm), at 37 °C. Results for ORAC were expressed as µmol of Trolox antioxidant equivalent per g of sample.

#### 4.3.3. FRAP Assay

Ferric-reducing ability power (FRAP) assay was done as described previously [11,31], with slight modifications. It is based on the antioxidant capacity of the sample in reducing iron present in the reaction. Calibration curve was in the range of 50 to 600 µmol × L^−1^ Fe (II). The values were expressed as µmol of Fe (II) sulfate equivalents per g of sample.

#### 4.3.4. Total Phenolic Content

Content of total phenolic compounds in the extracts was determined by the Folin-Ciocalteu reagent assay [32]. Calibration curve was in the range 20 to 100 mg × L^−1^ Gallic acid in 7% acetone. Total phenolics were expressed as Gallic acid equivalents (mg GAE × 100 g^−1^ extract).

#### 4.3.5. Total Flavonoid Content

Was determined as previously described [33]. Calibration curve was in the range 1 to 50 mg × L^−1^ of catechin. Total flavonoids were expressed as catechin equivalents (mg CE × 100 g^−1^ extract).

#### 4.3.6. Total and Monomeric Anthocyanin Content

Total anthocyanins were determined by the pH differential method [34]. The content of total and monomeric anthocyanins was expressed as mg cyanidin 3-glucoside equivalents 100 × g^−1^ extract. Content of total phenolic compounds in the extracts was determined by the Folin-Ciocalteu reagent assay [31]. Calibration curve was in the range 20 to 100 mg × L^−1^ Gallic acid in 7% acetone. Total phenolic were expressed as Gallic acid equivalents (mg GAE × 100 g^−1^ extract).

#### 4.3.7. Anthocyanins Profile in GPE by HPLC-PDA

The liquid chromatography system (Shimadzu^®^, Kyoto, Japan – they are all Shimadzu. Do we need to repeat the same information?) included a quaternary pump LC-20AT, PDA SPD-M20A, system controller CBM-20A, and degasser DGU-20A5. Chromatographic separation of anthocyanins was achieved using a reversed-phase column (C18, 5 µm, 250 mm × 4.6 mm, Kromasil^®^ (Bohus, Sweden). The mobile phase consisted of a gradient of 1% aqueous formic acid (Eluent A), 1% formic acid in methanol (Eluent B) and acetonitrile (Eluent C), at a flow rate of 1.0 mL/min. Eluent C concentration was kept constant at 2% during analysis. Prior to injection, the column was equilibrated with 22% B. After sample injection, solvent composition was kept constant until 10 min, increased to 45% B at 35 min and was kept constant until 50 min. Between injections, 10 min intervals were allowed to re-equilibrate the column with 22% B. The eluent was monitored between 200 and 600 nm, for spectra acquisition, and 530 nm was used for calculation of anthocyanins concentrations. Chromatographic peaks identities were based on retention time data and UV absorption spectra of commercial standards, and standard spiking. In cases of standards unavailability, peak identity designation was tentative and based on published data of Pinot noir anthocyanins composition by reversed-phase HPLC and anthocyanins published PDA-Vis-spectra [35,36,37]—those were the methods that were published, and not our results. Five-point calibration curves of delphinidin-3-glucoside and cyanindin-3-glucoside were done, from 1 to 10 mg × L^−1^ (*R*^2^ = 0.9925 and 0.9934, respectively, and *p* < 0.001). Petunidin-3-glucoside, peonidin-3-glucoside, and malvidin-3-glucoside were quantified as cyanindin-3-glucoside equivalents, using its calibration curve.

### 4.4. Cellular Assays

#### 4.4.1. Cell Culture

HepG2 and BEAS cell lines (from ATCC, Manassas, VA, USA), respectively of human hepatocarcinoma and normal human fibroblast, were grown in cell culture medium (respectively, DMEM and RPMI; Invitrogen, Waltham, MA, USA) supplemented with 10% fetal bovine serum (Invitrogen, Waltham, MA, USA) and maintained at 37 °C and 5% CO_2_. No antibiotics were used for cultivating the cells.

Control cells were incubated with matching volumes of 30% ethanol, in order to achieve the same final ethanol concentration found in the GPE.

#### 4.4.2. Cytotoxicity Assays

Three methods were used to evaluate the cytotoxicity of GPE: the MTT (3-(4,5-dimethylthiazol-2-yl)-2,5-diphenyltetrazolium bromide) and resazurin reduction and lactate dehydrogenase (LDH) assays. The MTT and resazurin assays evaluate the cellular metabolic activity (mainly mitochondrial dehydrogenases activity) and the LDH activity measured in the culture medium is a proxy of cellular membrane integrity. Briefly, cells were seeded in a 12-wells plate and incubated with grape pomace extract, at 75.0, 150.0 and 300.0 μg × mL^−1^, for 1, 5 or 24 h. After the incubation period, culture medium was removed, and cells were incubated with 0.5 mg × mL^−1^ MTT for 2 h at 37 °C. Subsequently, medium was aspirated and acid-isopropanol solution (0.1 N HCl) was added to all wells and mixed thoroughly to dissolve formazan crystals. The plate was placed in a microplate reader and the absorbance was measured at 570 nm and 650 nm (reference). Resazurin assay was done according to the manufacturer’s instructions (Promega Corporation, Madison, WI, USA) and was used to calculate the IC50 with the GPE concentration at 37.5, 75.0, 150.0, 300.0, 600.0, 900.0 μg × mL^−1^ for 24 h.

For the LDH assay the cells were incubated in a short-period (15, 30, 45 and 60 min) with GPE at 75.0, 150.0 and 300.0 µg × mL^−1^ and with GPE at 300.0 μg × mL^−1^ for 1, 5 or 24 h. Lactate dehydrogenase release was determined by using a LDH detection kit (Promega CytoTox 96 assay kit). The procedures were performed according to manufacturer’s instructions.

#### 4.4.3. Cell Death by Flow Cytometry

Cell death index was measured using a double staining method with The Vybrant Apoptosis Assay Kit # 2 (Molecular Probes, Eugene, OR, USA). HepG2 cells were incubated with grape pomace extract, at 75.0, 150.0 and 300.0 µg × mL^−1^, for 24 h. After incubation, cells were processed according to manufacturer’s instructions. Briefly, 10^6^ cells × mL^−1^ were centrifuged 5 min at 1200 RPM, 4 °C, and suspended in 100 μL Annexin V binding buffer. Subsequently, 5 μL of Annexin V-FITC and 10 μL of propidium iodide were added to each tube, except in the single stained controls, incubated for 15 min in the dark at room temperature. The samples were transferred to flow cytometer tubes in a final volume of 400 μL ice-cold Annexin V binding buffer. The acquisition and analysis were performed in the FACSCalibur flow cytometer using CellQuest software (BD Lifesciences, San Jose, CA, USA).

#### 4.4.4. Cellular Antioxidant Activity

Cellular antioxidant capacity was measured as described previously [38]. Human hepatocellular carcinoma HepG2 were seeded at a density of 10^4^ cells/well on a 96-well microplate. 48 h after seeding, growth medium was removed and the cells were washed with phosphate buffer. Triplicate wells were treated with grape pomace extract (75.0, 150.0 or 300.0 µg × mL^−1^) for 1 h and 50 μmol × L^−1^ CM-H_2_DCFDA was added 30 min before the end of the incubation period. DCFDH-DA oxidation was evaluated under cellular oxidative stress after antimycin A (2 mg × mL^−1^) addition, which is an inhibitor of mitochondrial complex III and a potent inducer of reactive oxygen species formation [39]. Therefore, the resulting effects of GPE on CM-H_2_DCFDA oxidation is a proxy of the cellular antioxidant capacity. Fluorescence was measured at 538 nm (emission) and 485 nm (excitation) every 5 min for 2 h in a microplate reader, at 37 °C (Victor X3, Perkin Elmer Inc., Waltham, MA, USA). Gallic acid was used as a reference antioxidant standard.

#### 4.4.5. Oxygen Consumption Analysis and Calculation of Respiratory Parameters

Mitochondrial oxygen consumption rates were monitored and evaluated by high-resolution respirometry with Oxygraph-2 k (Oroboros Instruments, Innsbruck, Austria). This instrument provides sufficient sensitivity to detect subtle changes in cellular respiration and allows the utilization of small sample size. Oxygen consumption rates were measured in intact HepG2 cells, suspended in the culture medium (DMEM, 5 mmol × L^−1^ glucose) with fetal bovine serum, at cell density of 2.0 × 10^6^ cell × mL^−1^, at 37 °C in 2 mL chamber, at stirring rate of 750 rpm, as described by us previously [27]. Briefly, cells were harvested with trypsin and washed twice with complete media. Cells were counted, and viability checked with trypan blue dye and suspended in complete culture media. In each experiment, oxygen consumption rates were determined in a time interval up to 1 h. After cells were added to the respiration chamber, routine respiration (Routine) was measured. Routine respiration reflects the rate of oxygen consumption in the presence of respiratory substrates glucose (5 mmol × L^−1^) + glutamine (2 mmol × L^−1^), in the coupled state, i.e., when oxidative phosphorylation is active. Subsequently, 2 consecutive additions of GPE (300.0 µg × mL^−1^, each) were performed. After constant respiratory flux, 1 μg × mL^−1^ oligomycin was added and Uncoupled respiration (Uncoupled) was recorded. Oligomycin inhibits mitochondrial phosphorylation system and Uncoupled respiration corresponds to oxygen consumption uncoupled to ATP synthesis. The difference Routine-Uncoupled respirations corresponds to Coupled respiration, i.e., oxygen consumption coupled to ATP synthesis. The maximum uncoupled respiration was measured in the presence of optimum carbonyl cyanide *p*-(trifluoromethoxy) phenylhydrazone (FCCP) concentration (200 nmol × L^−1^). Optimum FCCP concentration was determined after FCCP titration (40–500 nmol × L^−1^, data not shown). Maximal uncoupled respiratory activity is a measure of Electron Transport System (ETS) capacity. Since this was recorded on intact cells, it reflects ETS capacity under physiological substrate supply [40].

ETS capacity was used to normalize and calculate respiratory flux control ratios. Routine flux control ratio (Routine/max) reflects mitochondrial activity related to maximal ETS capacity and corresponds to how much spare respiratory capacity the cells present [41]. Uncoupled flux control ratio (Uncoupled/max) reflects Uncoupled respiration as a function of ETS and corresponds to the extent of intrinsic uncoupling. Finally, Coupled/max ratio, which is calculated by (Routine-Uncoupled)/Maximal, is the fraction of ETS capacity used to drive ATP synthesis [27]. Data acquisition and analysis were done with DatLab 4.3 software (Oroboros Instruments, Innsbruck, Austria).

#### 4.4.6. Glucose Uptake and Lactate Efflux to the Culture Medium

Glucose uptake from and lactate efflux to the culture media were evaluated by Nuclear Magnetic Resonance (NMR). After 1 h incubation of HepG2 cells with GPE, culture medium was replaced with fresh DMEM (no glucose) supplemented with 5 mmol × L^−1^ D-[U-13C] glucose, as described previously [42]. At a 15 min intervals (0–180 min), 0.5 ml aliquots were collected from the culture medium to evaluate glucose disappearance from and lactate efflux to the culture medium. The same volume of medium was replaced to achieve a constant D-[U-13C] glucose “pulse” during the experiment. Antimycin A (2 mg × mL^−1^), which inhibits mitochondrial respiration, was added to cells to evaluate the effects of GPE when HepG2 cells relied solely on anaerobic glycolysis for ATP production. This approach makes it possible to evaluate the effects of GPE on both glycolytic capacity and mitochondrial function. To evaluate the kinetics of glucose uptake and lactate efflux, one-dimensional ^13^C spectra were obtained at 298 K, using 45° pulses with a repetition time of 0.6 s, 16,000 complex points, 2028 scans and a spectral width of 200 p.p.m. The free-induction decays were zero-filled to 16,384 points and apodized with exponential multiplication using line broadening of 10 Hz. Spectra were acquired with a Bruker DRX 400 MHz (Bremen, Germany) using a triple resonance probe (TXI). Spectral processing and analysis was performed using Topspin 3.5 (Bremen, Germany). Analysis and assignment of glucose and lactate were obtained using the Human Metabolome Database v 3.6 [43]. Glucose and lactate in the samples were quantified by their peak areas.

#### 4.4.7. Nuclear Magnetic Resonance (NMR) for Analysis of Intracellular Metabolites

Intracellular metabolites were evaluated by Nuclear Magnetic Resonance (NMR). After 1 h incubation of HepG2 cells with GPE, cells were washed twice with PBS and intracellular metabolites were extracted using a mixture of chloroform:methanol as previously described [44]. Approximately 5 × 10^7^ cells (50 mg protein) were used in each extraction and the polar/methanolic phase was freeze dried and kept on −80 °C until analysis. Dried extracts were suspended in 100 mmol × L^−1^ phosphate buffer, pH 7.4, 100% deuterium oxide and 0.1 mmol × L^−1^ of 4,4-dimethyl-4-silapentane-1-sulfonic acid (DSS) for reference. NMR spectra were collected in Bruker Avance III HD (Bremen, Germany) operating at 500.13 MHz for ^1^H at 298 K. ^1^H spectra were acquired with excitation sculpting for water saturation, 12.9836 ppm spectral width, 1.74 s relaxation delay, 32 K points and 3 K accumulations. ^1^H-^1^H TOCSY spectra were used for metabolites assignments. Spectra were processed in TOPSPIN 3.5 (Bruker-Biospin, Bremen, Germany). For comparison between GPE-treated and control HepG2 cells, the peaks integrals were calculated. For statistical sampling, we collected datasets for 3 independent cellular extracts.

### 4.5. Statistical Analysis

Descriptive statistics, mean and standard error, were calculated for all data. Student’s *t* tests and analysis of variance (ANOVA) with Tukey’s post-test were used to compare mean values. Differences were considered to be statistically significant when *p* < 0.05. All analyses were performed with GraphPad Prism 7 software (La Jolla, CA, USA).

## 5. Conclusions

In the present study, we showed that Pinot noir grape pomace extract from an industrial white winemaking process, concentrated by membrane technology in a pilot plant scale, induced early metabolic changes on human hepatocarcinoma HepG2 cells. Metabolic effects seemed somehow targeted to mitochondria and preceded the observed decrease on cell proliferation and viability. These results indicate that GPE present important pharmacological properties, with potential future use as functional and nutraceutical food ingredients. Future investigations of the cellular mechanisms of action of GPE and in vivo studies are needed to support its nutraceutical use in humans.

## Figures and Tables

**Table 1:** Total Phenolics and flavonoids, total and monomeric anthocyanins, and antioxidant capacity of grape (*Vitis vinifera* L. cv Pinot Noir) pomace extract, by spectrophotometric assays.

Phenolic Compounds (Mean ± SE; mg × 100 g^−1^ DM ^a^)				Antioxidant CAPACITY (Mean ± SE; µmol × L^−1^ × g^−1^ DM)		
TPC ^b^	Total Flavonoids ^c^	Total Anthocyanins ^d^	Monomeric Anthocyanins ^d^	ABTS ^e^	FRAP ^f^	ORAC ^e^
34,061 ± 860.1	2146 ± 110.6	1582 ± 7.4	1441 ± 8.2	1014 ± 0.1	3220 ± 6.4	167.1 ± 1.0

^a^ DM, dry matter; ^b^ total phenolic compounds, expressed as gallic acid equivalents; ^c^ expressed as catechin equivalents; ^d^ expressed as cianidin 3-glucoside; ^e^ expressed as trolox equivalent; ^f^ expressed as Fe(II) sulfate equivalents. *n* = 5 independent experiments.

## Supplementary Materials

The following are available online. Figures S1 to S4: PDA-Visible spectra of anthocyanins found in GPE; Figure S5: Cytotoxicity of grape pomace extract (GPE) on human hepatocarcinoma cell line HepG2; Figure S6: Grape pomace extract is not cytotoxic to BEAS human fibroblast cell line.
