# Supplementary material for: Anthocyanin-Rich Grape Pomace Extract (Vitis vinifera L.) from Wine Industry Affects Mitochondrial Bioenergetics and Glucose Metabolism in Human Hepatocarcinoma HepG2 Cells
Source: Molecules. 2018 Mar 8;23(3):611. doi: 10.3390/molecules23030611 (PMC6017946; doi:10.3390/molecules23030611)
Supplement: Supplementary file 1 [file molecules-23-00611-s001.zip › Sales_etal_SupplementaryFig6.pptx]

## Slide 1
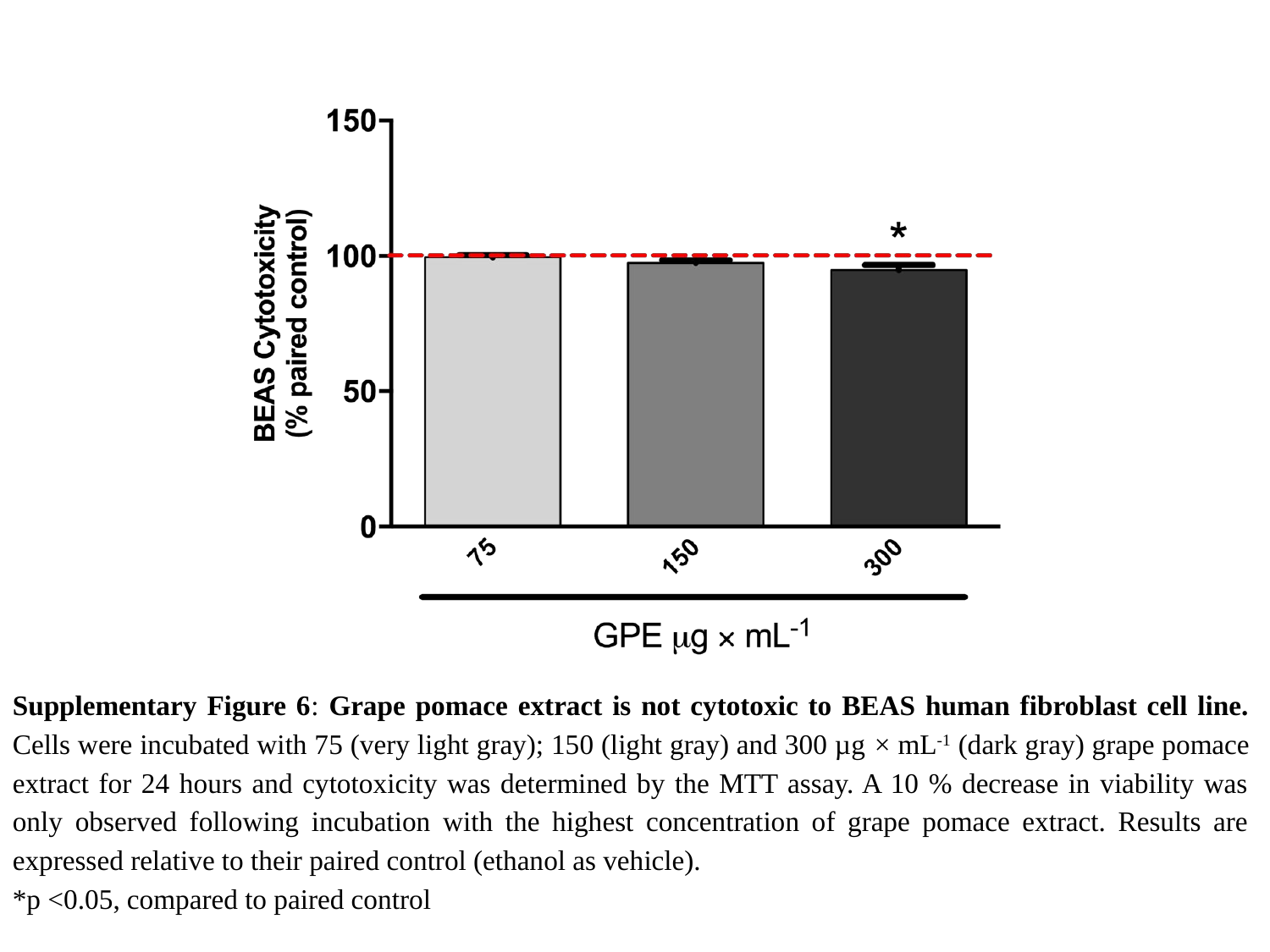

Supplementary Figure 6: Grape pomace extract is not cytotoxic to BEAS human fibroblast cell line. Cells were incubated with 75 (very light gray); 150 (light gray) and 300 µg × mL-1 (dark gray) grape pomace extract for 24 hours and cytotoxicity was determined by the MTT assay. A 10 % decrease in viability was only observed following incubation with the highest concentration of grape pomace extract. Results are expressed relative to their paired control (ethanol as vehicle).
*p <0.05, compared to paired control
