# Supplementary figures and images for: Anthocyanin-Rich Grape Pomace Extract (Vitis vinifera L.) from Wine Industry Affects Mitochondrial Bioenergetics and Glucose Metabolism in Human Hepatocarcinoma HepG2 Cells
Source: Molecules. 2018 Mar 8;23(3):611. doi: 10.3390/molecules23030611 (PMC6017946; doi:10.3390/molecules23030611)

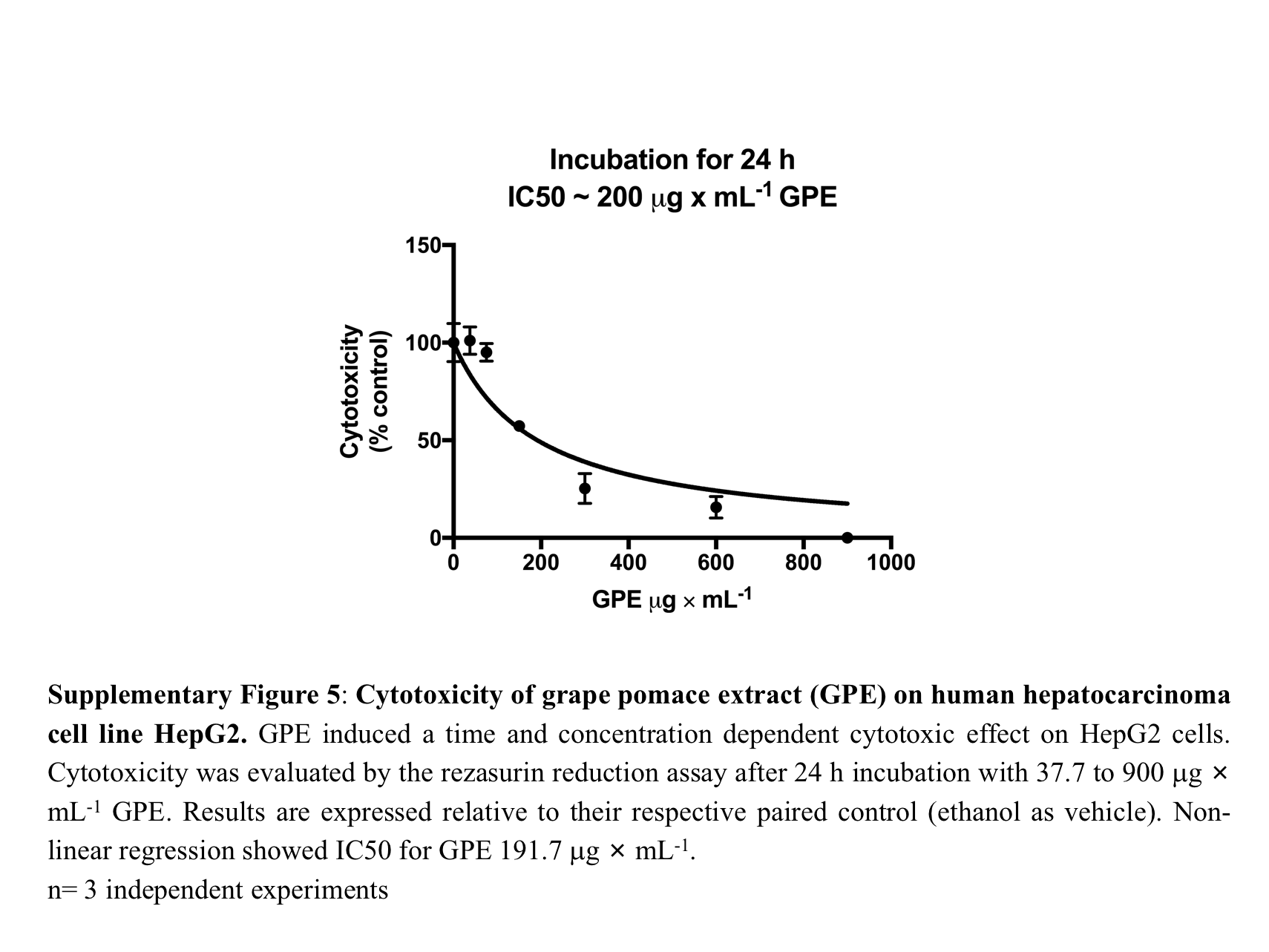

Supplement: Supplementary file 1 [file molecules-23-00611-s001.zip › Sales_etal_SupplementaryFig5.tiff]

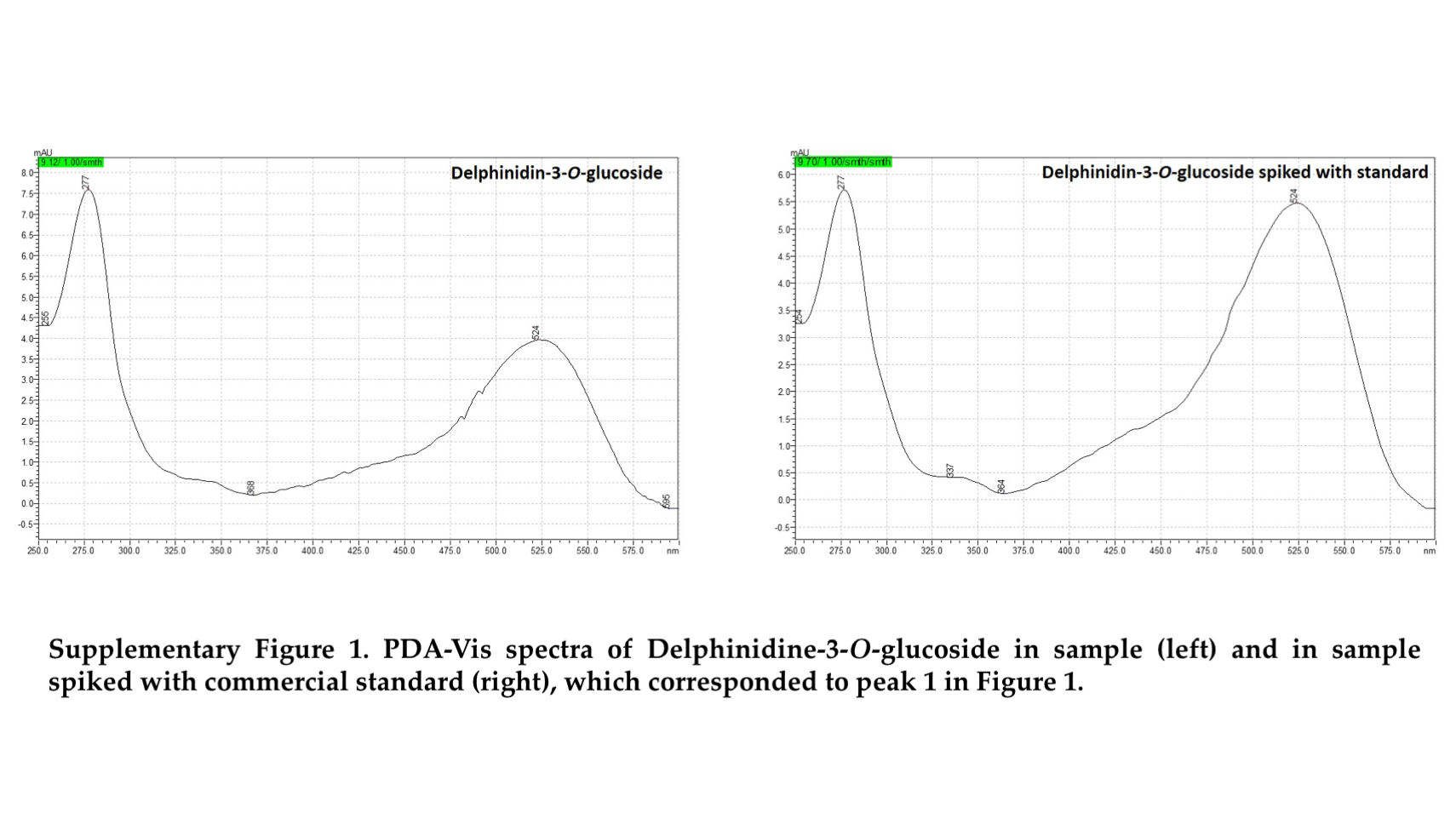

Supplement: Supplementary file 1 [file molecules-23-00611-s001.zip › Sales_etal_SupplementaryFigure1.tiff]

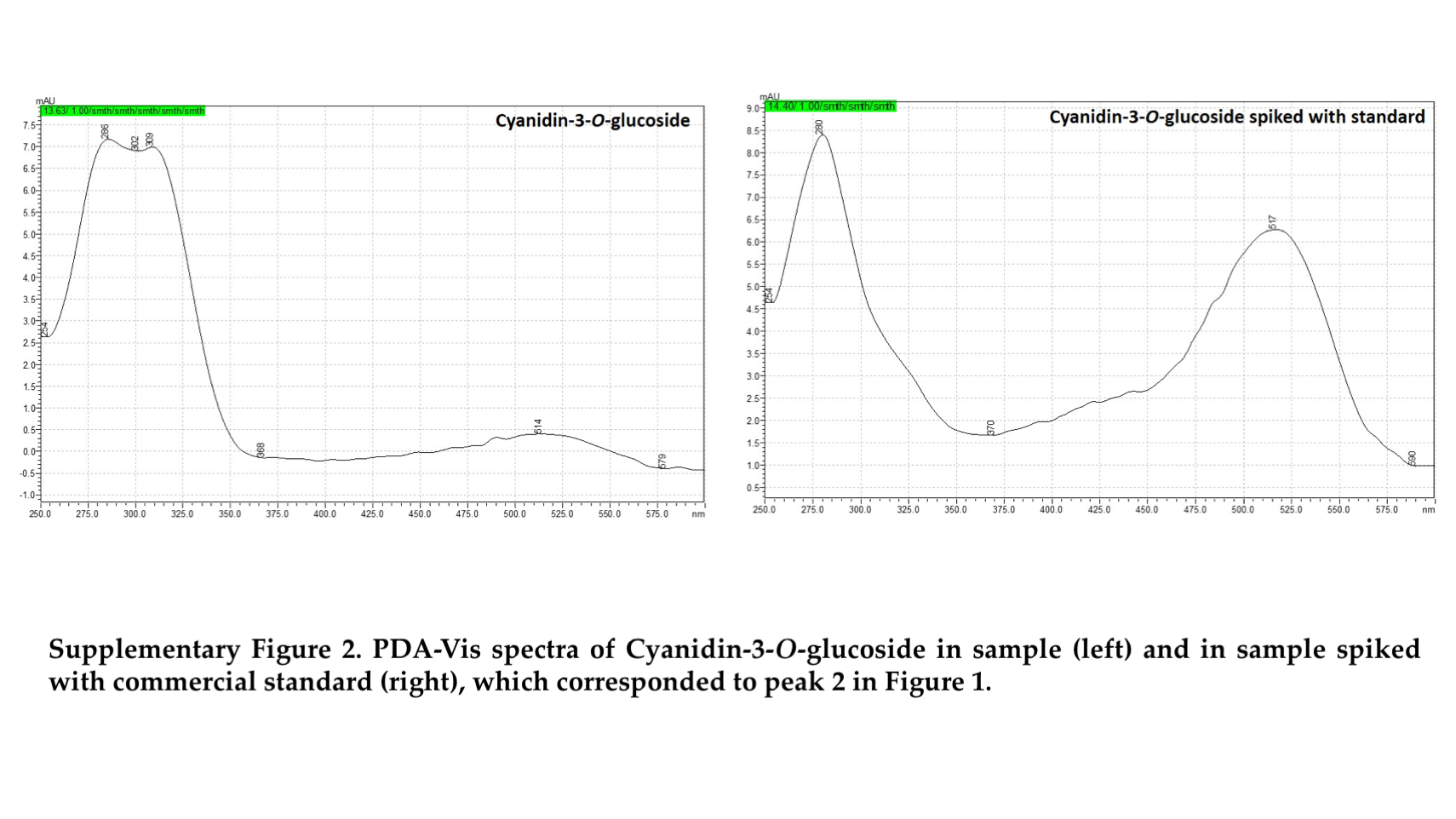

Supplement: Supplementary file 1 [file molecules-23-00611-s001.zip › Sales_etal_SupplementaryFigure2.tiff]

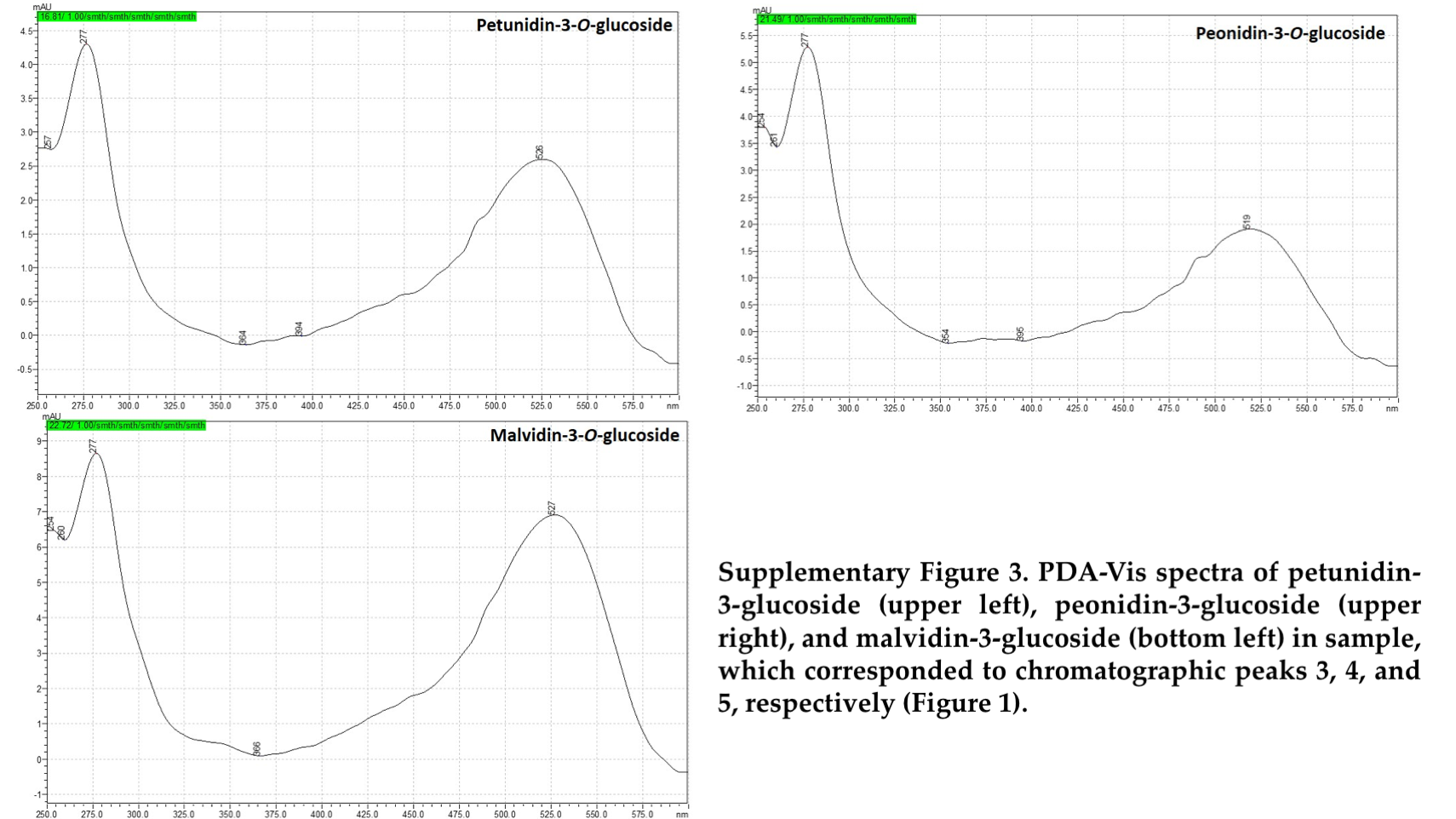

Supplement: Supplementary file 1 [file molecules-23-00611-s001.zip › Sales_etal_SupplementaryFigure3.tiff]

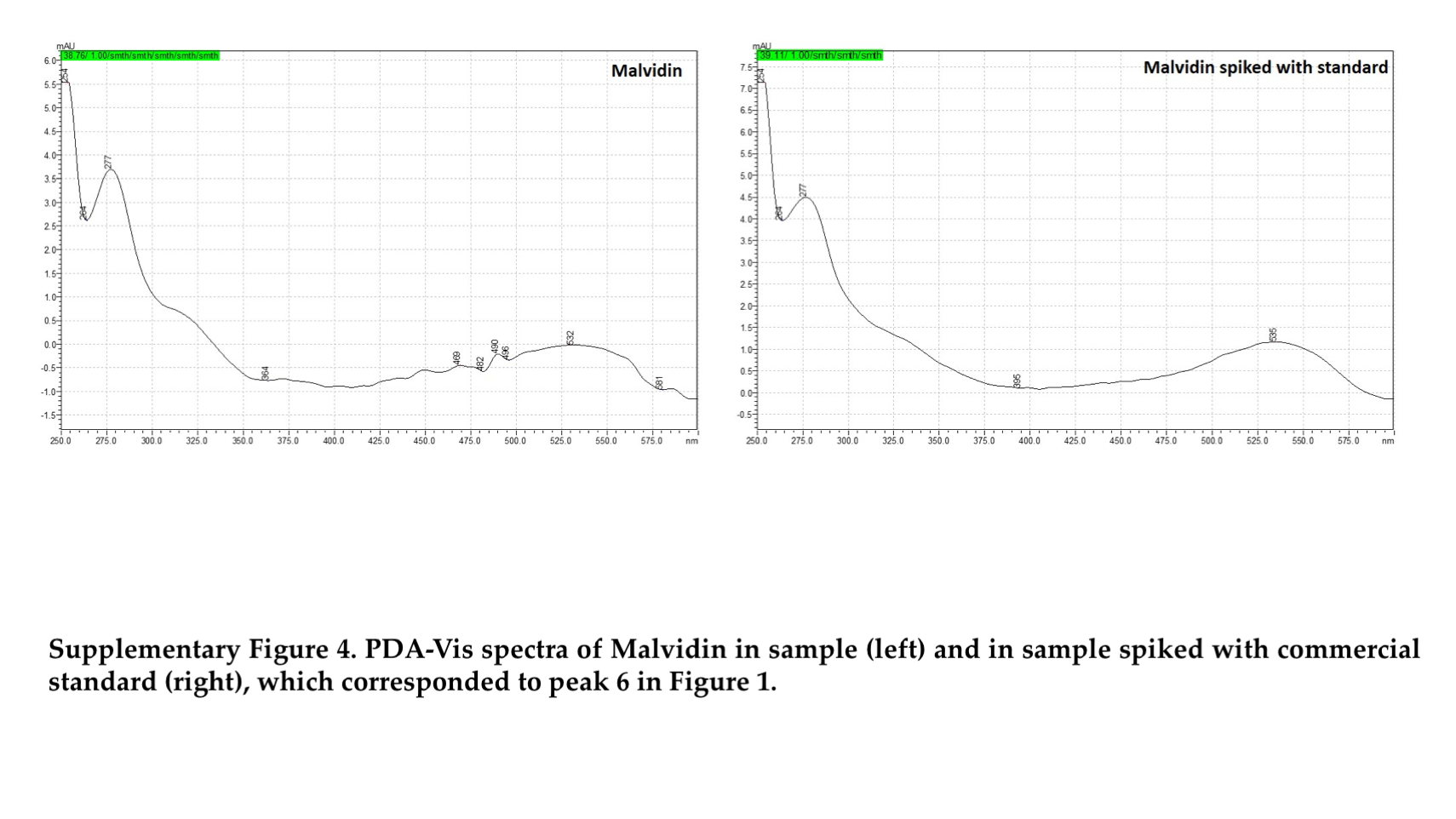

Supplement: Supplementary file 1 [file molecules-23-00611-s001.zip › Sales_etal_SupplementaryFigure4.tiff]
